# Advancements in the Ocular Prosthesis Technology: The Insightful Innovations

**DOI:** 10.7759/cureus.66409

**Published:** 2024-08-07

**Authors:** Vedant Pathak, Seema Sathe, Anjali Bhoyar, Surekha A Dubey, Tanvi Jaiswal, Arushi Beri

**Affiliations:** 1 Prosthodontics, Sharad Pawar Dental College and Hospital, Wardha, IND

**Keywords:** custom, digital, photograph, eye, prosthesis, ocular, iris positing, rehabilitation, occular prosthesis

## Abstract

In patients who have lost their eye due to one or many of the following events, such as surgery, trauma, tumors, or birth eye defects, ocular prostheses play a very vital role in the recovery of their eye appearance and social appearance. This case report highlights the successful fitting and fabrication of a custom-made ocular prosthesis in a patient with an eye defect. The manufacturing process of the prostheses involved careful evaluation and fabrication involving a series of steps to achieve a successful prosthesis. A customized prosthesis guarantees excellent esthetic matching, more comfort, and increased patient confidence, thereby a better quality of life.

## Introduction

An individual's social, mental, and physical well-being is grossly affected due to the loss of an eye [1]. Some causes include tumors, congenital anomalies, irreversible trauma, and sympathetic ophthalmia [2]. Following are the three major surgical techniques for the removal of an eye [2]: (1) enucleation - the removal of the entire globe, including the sclera, intraocular contents, and the cornea. The stump of the optic nerve as well as the extraocular muscles are left behind. (2) Evisceration - the removal of intraocular contents including the lens, uvea, retina, vitreous humor, and in some cases the cornea. Only the sclera and extraocular muscles remain intact. (3) Exenteration - the removal of the globe and all of the orbital contents. This procedure may include the removal of selective sections of orbital bone [3].

Ocular prostheses provide practical solutions by restoring the natural appearance of the eye, giving a feeling of normality, and enhancing the quality of life and self-esteem [3]. Following are the two types of prosthesis that are in use: the prefabricated and the custom-made. Prefabricated prostheses are available in standard sizes, shapes, and colors; customized ones distribute pressure evenly, fit well, and give better esthetics [4-10]. Examples of biocompatible materials that are normally used for ocular prostheses include medical-grade acrylics and silicone elastomers. The medical-grade acrylic is used to replace the eyeball, whereas silicone elastomers are used to replace surrounding soft tissue structures that surround the eyeball (i.e., eyelid, etc.). The choice of material depends on the extent of structures that have to be replaced [7,10]. These materials are strong, long-lasting, and impervious to bacteria and moisture. Thanks to advancements in fabrication techniques (such as computer-aided design and three-dimensional printing), the production of customized ocular prostheses has become more accurate and productive. This study outlines a straightforward, reasonably economical method for creating an acrylic ocular prosthesis by using the grid method for iris positioning.

## Case presentation

**Figure 1:**
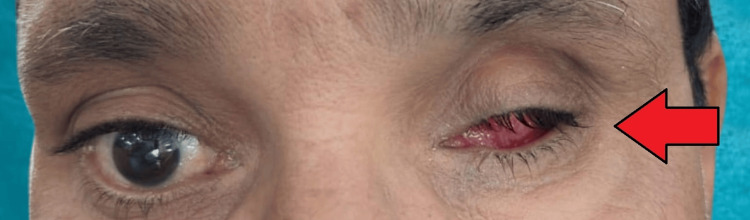
Defect of the left eye after enucleation six months ago.

A 50-year-old male patient reported to the prosthodontics department to treat his enucleated left eye. On record, the medical history of the patient revealed that he had to undergo surgical enucleation of his left eye due to trauma about six months ago (Figure 1). On examination of the affected eye, the eye was sunken and a healed bed was seen.

**Figure 2:**
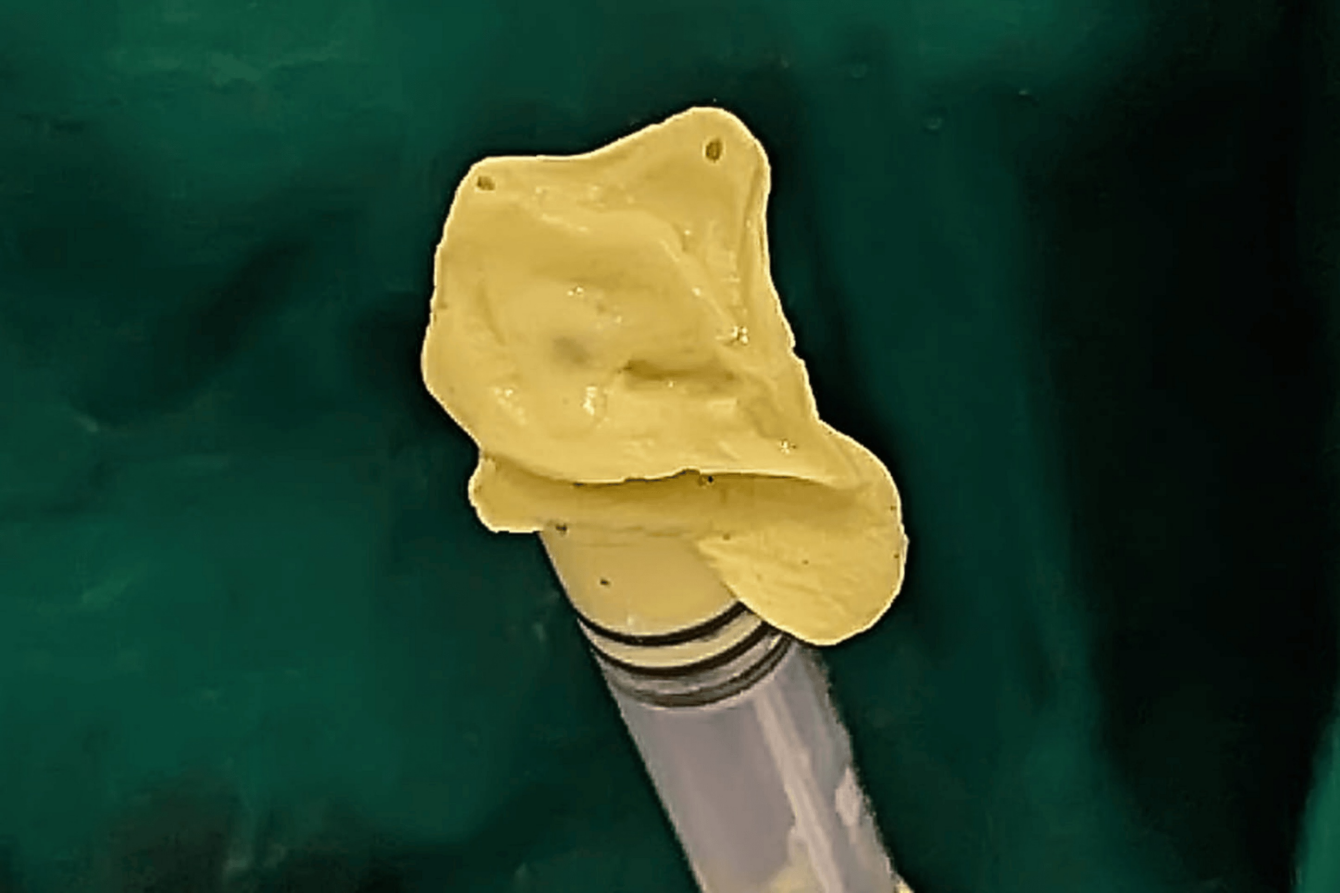
Preliminary impression of the defect with irreversible hydrocolloid impression material.

The procedure for fabricating the customized eye began with taking a preliminary impression using irreversible hydrocolloids. Before taking impression, the patient's eye was irrigated with a normal saline solution, followed by the application of petroleum jelly. Irreversible hydrocolloid impression material was used to record the preliminary impression by injecting it into the eye socket with a syringe (Figure 2). The patient was instructed to carry out movements of the eyes to record the impression in a functional state.

**Figure 3:**
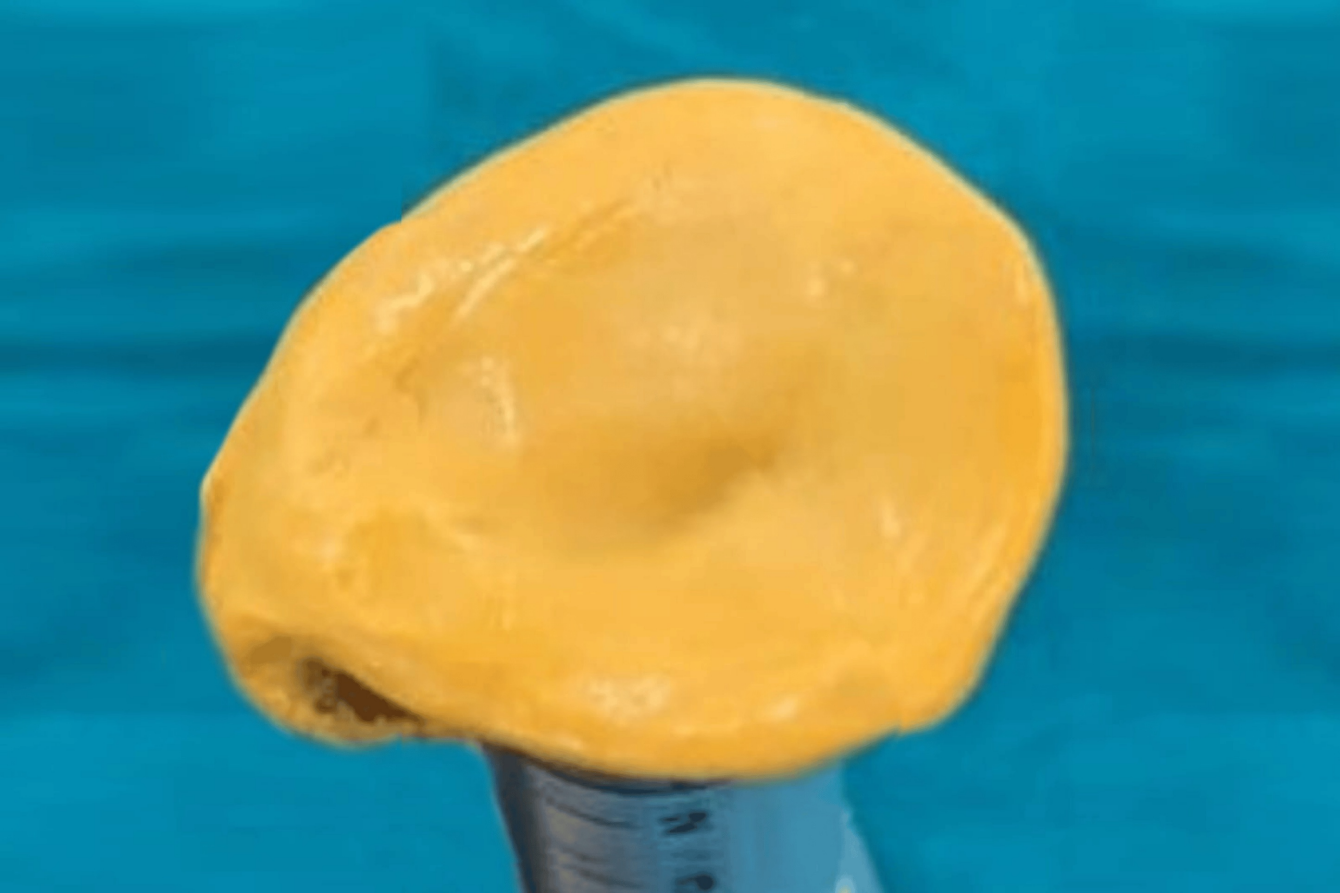
Final impression of the left eye using light body wash impression material.

The impression was poured into type 3 gypsum, and a custom tray was fabricated using clear acrylic resin. The tray was then attached to a syringe, ensuring it was firmly secured. Finally, the final impression of the left eye was obtained using the special tray and light body wash impression material (Figure 3).

**Figure 4:**
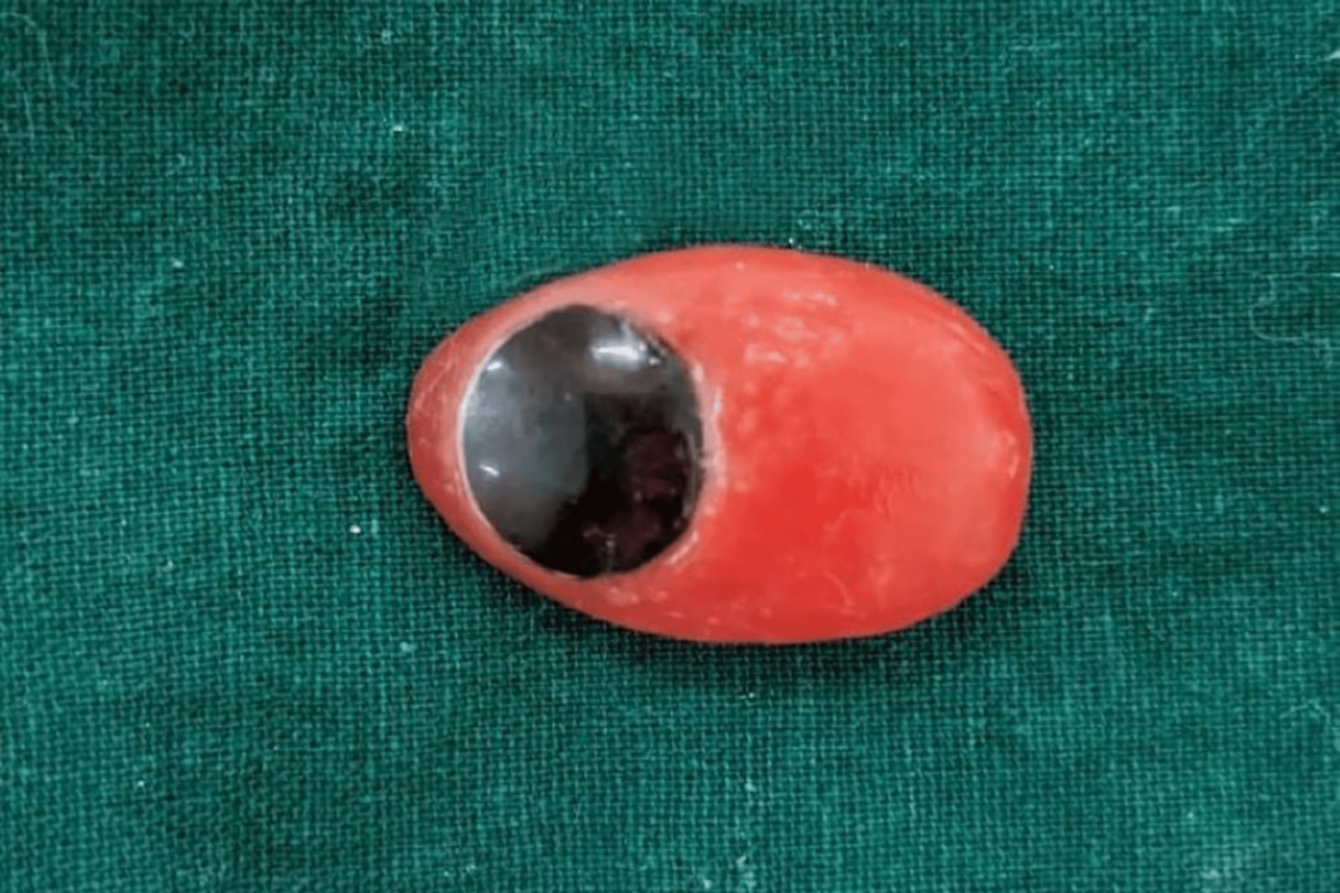
Wax pattern of the prosthesis with tentative iris positioning after shade selection and matching with normal eye.

After obtaining the impression, the cast was poured to prepare the mold. A wax pattern was fabricated on the cast. Figure 4 illustrates the fabricated wax pattern with the iris positioned tentatively. 

**Figure 5:**
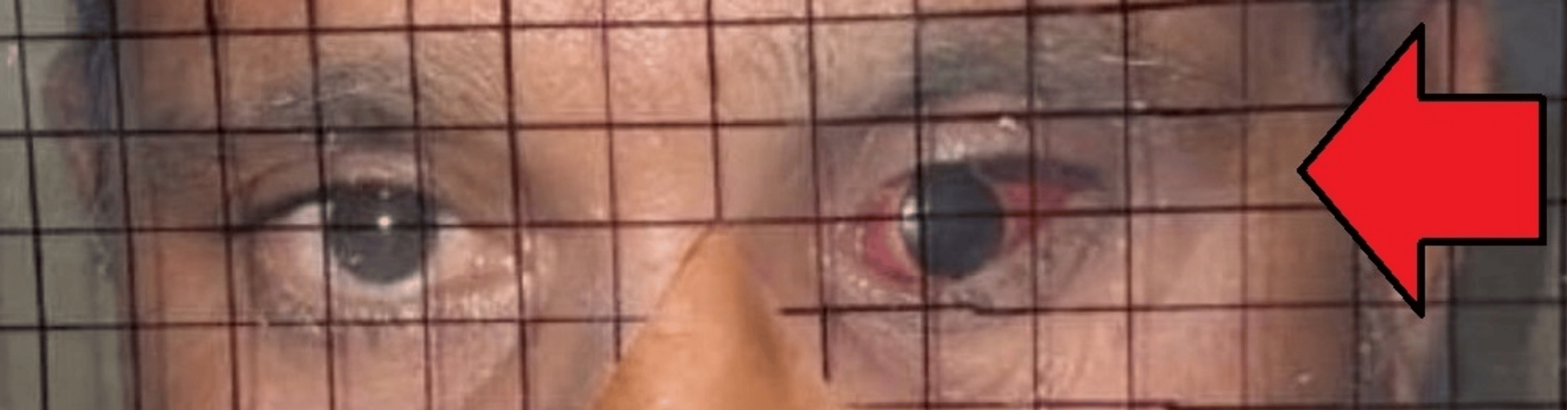
Grid used for definitive iris positioning of the prosthesis.

The wax pattern was inserted into the eye socket and verified for contour and functions, such as closing and opening of the eye. To verify the relative location of the iris, a grid made from clear cellophane paper with evenly spaced markings was used. The measurement from the midline to the natural position of the iris was recorded and used to position the iris in the affected left eye. The patient was asked to perform various eye movements to ensure the iris was correctly positioned (Figure 5).

**Figure 6:**
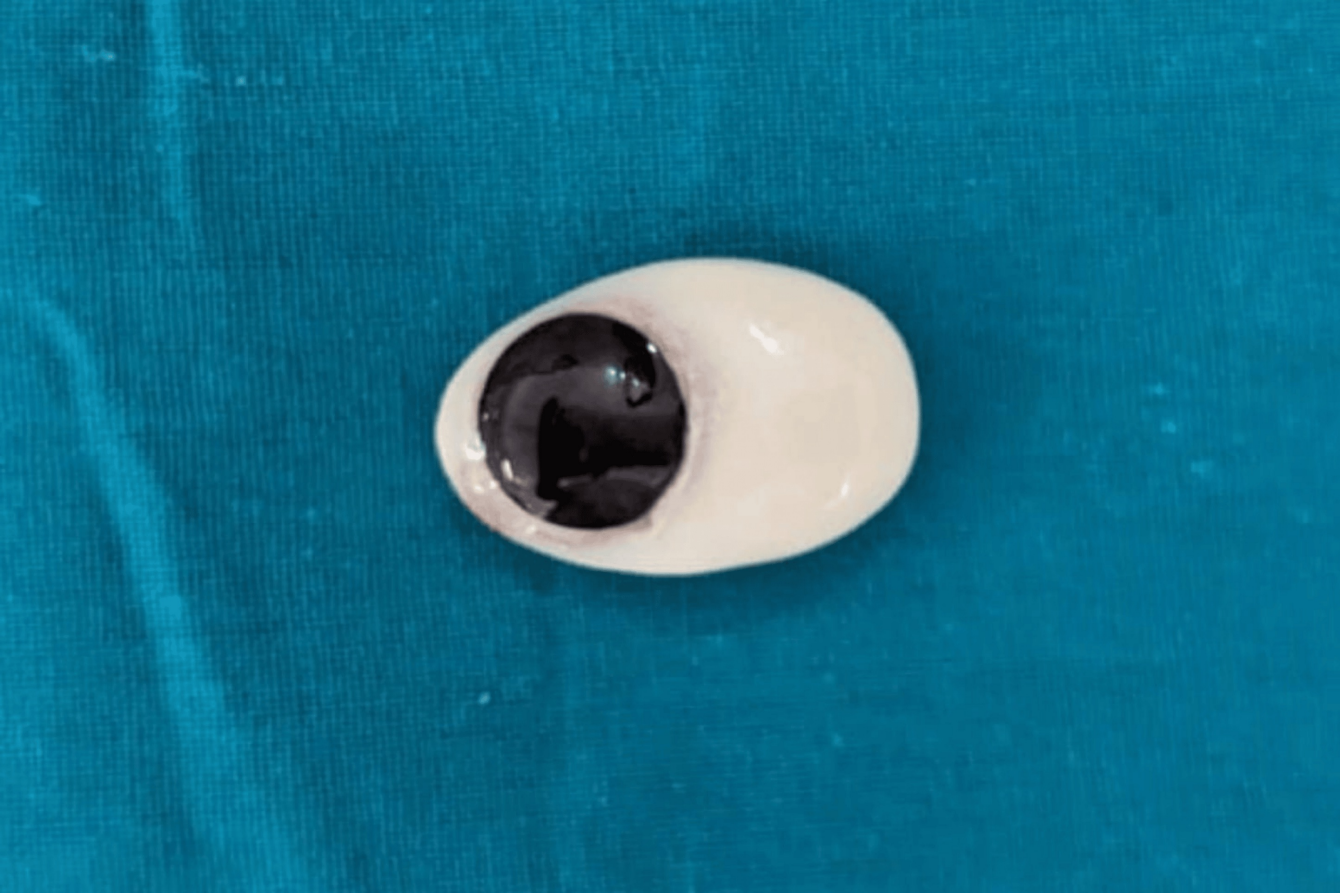
Final prosthesis after finishing and polishing of the prosthesis.

**Figure 7:**
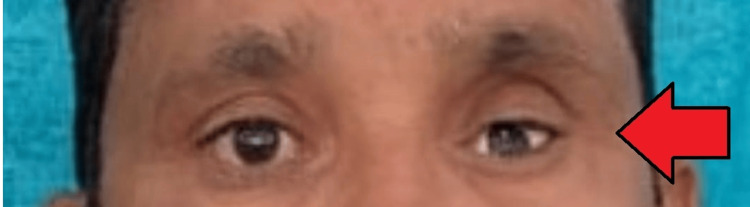
Placement of the prosthesis in the site to be matched with normal eye with respect to dimension of the prosthesis.

**Figure 8:**
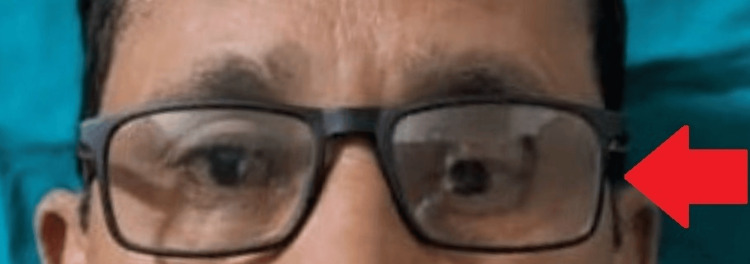
Patient with spectacles for enhanced esthetic.

After confirming the iris position, a sprue was attached to maintain its alignment during packing. The packing was done with heat-cured acrylic resin, following the manufacturer's instructions for conventional curing. The ocular prosthesis was then carefully polished and finished. Before insertion into the socket, the prosthesis was cleaned and lubricated with an ocular lubricant to ensure smooth eye movement (Figure 6). After giving instructions on the care and maintenance of the prosthesis, it was delivered to the patient (Figure 7). The patient was also advised to wear spectacles for improved esthetics and to protect the prosthesis (Figure 8).

## Discussion

Owing to its complex architecture and vital role in vision, the eye is a necessary sense organ of life. Individuals who lose their vision due to various reasons face both psychological and physical challenges [2]. The iris placement is the most important step in the creation of a prosthetic eye. A properly positioned iris adds symmetry and realism to the patient's face. Using a wide range of techniques and methods, the precise location of the iris has been widely established in the literature [3]. Subjective procedures include the use of an ocular locator, grids, fixed calipers, divisions, visual evaluation, and inverted anatomic tracings. To position the iris precisely, these methods manipulate the operator's perception. We used the grid method for positioning the iris in our case, as it is the most widely accepted method that precisely orients the iris to match the contralateral eye.

A combination of technical and artistic skills is needed for the intricate process of fabricating an ocular prosthesis. A few methods to speed up and simplify the fabrication process have been published in the literature. Many authors have recommended making a custom tray for ocular impressions [4-10]. In this instance, creating the personalized tray was an easy and quick workaround. To obtain a good fit, it has also been recommended to modify an existing stock or custom ocular prosthesis using relining material [6]. Ocular prostheses based on polymethyl methacrylate (PMMA) have been widely used by numerous authors [7]; hence, this was used in our case. It has also been suggested to incorporate a porcelain sclera veneer; however, this alternative is a costly and time-consuming procedure [9].

Due to its weight reduction and flexibility over acrylic, medical-grade silicone has been recommended [10]. Acrylic was used in this instance for its strength, translucency, and affordability. The two options for matching the patient's neighboring eye are painting the iris or adding a stock iris [7]. In one case, digital photography was used to assist in creating a duplicate iris along with the grid method. A clear co-polyester sheet was vacuum-pressed onto the photo paper, as described by Kale et al., who produced digital representations of the iris and sclera [8]. The technique accurately replicated the patient's natural eye despite being challenging and time-consuming to master. The process outlined in this instance was reasonably priced, easy to use, and effective [11]. However, we used the stock eye, and after matching the iris, the sclera was trimmed and replaced. By carefully recreating the patient's contralateral natural eye using a photograph of the iris and applying oil paints to the sclera for shade matching, we were able to achieve a consistent result.

## Conclusions

The precise orientation of the iris is crucial for the success of an ocular prosthesis. To achieve the best esthetic outcomes, it is recommended to use objective iris-positioning techniques that rely on a basic toolkit and do not require rigorous patient cooperation or assistance. This involves having a thorough understanding of each technique and staying informed about upcoming developments. Customizing an ocular prosthesis is a challenging process that demands accuracy and precision. This study describes an advanced technique that helps clinicians save time and effort in creating custom ocular prostheses. This method ensures a secure and comfortable fit while maintaining a natural appearance.
